# Adiponectin Deficiency Blunts Hypoxia-Induced Mobilization and Homing of Circulating Angiogenic Cells

**DOI:** 10.1155/2013/260156

**Published:** 2013-10-29

**Authors:** Bert R. Everaert, Vincent J. Nijenhuis, Florence C. M. Reith, Vicky Y. Hoymans, Jean-Pierre Timmermans, Christiaan J. Vrints

**Affiliations:** ^1^Laboratory of Cell Biology and Histology, University of Antwerp, Groenenborgerlaan 171, 2020 Antwerp, Belgium; ^2^Laboratory of Cellular and Molecular Cardiology, University Hospital Antwerp, Wilrijkstraat 10, 2650 Edegem, Belgium

## Abstract

*Aim*. We investigated the effects of adiponectin deficiency on circulating angiogenic cell (CAC) mobilization, homing, and neovascularization in the setting of acute myocardial infarction (AMI). *Methods & Results*. AMI was induced in wild-type (WT) (*n* = 10) and adiponectin knockout (*Adipoq* ^−/−^) mice (*n* = 7). One week after AMI, bone marrow (BM) concentration and mobilization of Sca-1^+^ and Lin^−^Sca-1^+^ progenitor cells (PCs) were markedly attenuated under *Adipoq* ^−/−^ conditions, as assessed by flow cytometry. The mRNA expression of HIF-1-dependent chemotactic factors, such as *Cxcl12* (*P* = 0.005) and *Ccl5* (*P* = 0.025), and vascular adhesion molecules, such as *Icam1* (*P* = 0.010), and *Vcam1* (*P* = 0.014), was significantly lower in the infarction border zone of *Adipoq* ^−/−^ mice. Histologically, *Adipoq* ^−/−^ mice evidenced a decrease in neovascularization capacity in the infarction border zone (*P* < 0.001). Overall, capillary density was positively correlated with Sca-1^+^ PC numbers in BM (*P* = 0.01) and peripheral blood (PB) (*P* = 0.005) and with the expression of the homing factors *Cxcl12* (*P* = 0.013), *Icam1* (*P* = 0.034) and *Vcam1* (*P* = 0.014). *Conclusions*. Adiponectin deficiency reduced the BM reserve and mobilization capacity of CACs, attenuated the expression of hypoxia-induced chemokines and vascular adhesion molecules, and impaired the neovascularization capacity one week after AMI.

## 1. Introduction

Cardiovascular disease (CVD) is a major cause of illness and death in industrialized countries [1]. Moreover, the increasing incidence of CVD is largely due to the global epidemic of obesity [2] and obesity-related comorbidities, such as diabetes mellitus [3]. As a consequence, the pathophysiological role of adipose tissues in the development of CVD has recently gained much attention.

Adipose tissue is nowadays considered to be an important endocrine organ [4], secreting a number of hormones, adipocytokines, which are of prime importance in the metabolic complications of obesity [5]. One specific adipocytokine, adiponectin, has been reported to offer cardiovascular protection in addition to its beneficial effects on insulin sensitivity. Some of the mechanisms, by which adiponectin exerts its antiatherogenic and anti-inflammatory properties, were reviewed by Goldstein et al. [6] and related to a reduction in the generation of reactive oxygen species (ROS), attenuation of the levels of proinflammatory cytokines, such as TNF*α*, and activation of endothelial nitric oxide synthase (eNOS). 

Clinically, adiponectin is inversely correlated with fasting plasma glucose and insulin levels and reduced in patients with type 2 diabetes mellitus and coronary artery disease [7]. Although higher adiponectin concentrations are usually associated with a favorable CVD risk profile, the value of adiponectin as a prognostic risk factor for the development or progression of CVD is currently still unclear. For instance, hypoadiponectinemia has been linked to early-onset coronary heart disease in men [8], and higher adiponectin levels have been found to be indicative of myocardial salvage after AMI [9]. Other research groups, however, reported that higher adiponectin levels are correlated with an increased 20-year CVD and all-cause mortality [10], all-cause mortality in patients with unstable angina referred for coronary angiography [11], or cardiovascular events in chronic heart failure patients [12]. These contradictory findings warrant further investigation into the value of adiponectin as a general CVD risk stratifier.

However, the effects of adiponectin on *in vitro* cell cultures and in *in vivo* knockout studies are more straightforward. In ischemia-reperfusion (IR) injury, adiponectin deficiency resulted in larger MIs, with increased myocardial cell apoptosis and a higher degree of inflammation [13]. These effects could be counteracted in *Adipoq* ^−/−^ mice by adiponectin supplementation [13, 14], which additionally attenuated cardiac remodeling after MI in WT mice. Adiponectin has also been reported to promote vessel growth *in vitro* and *in vivo *[15, 16], inhibit TNF*α*-induced nuclear factor *κ*B (NF*κ*B) signaling [17], and suppress endothelial cell apoptosis by upregulation of phosphorylated 5′ AMP-activated protein kinase (p-AMPK) [18]. Interestingly, the adiponectin-induced improvement in proliferation and migration of endothelial cells was similar to that observed with vascular endothelial growth factor (VEGF) [15]. Mechanistically, adiponectin activates the AMPK/PI3 K/AKT/eNOS pathway, resulting in upregulation of phosphorylated eNOS, which in turn increases NO production [14, 19]. 

Since eNOS also plays an important role in CAC biology [20], we hypothesize that downregulation of adiponectin affects CAC mobilization and peripheral homing after MI. Shibata et al. have already reported a decreased number of Sca-1^+^KDR^+^ circulating CACs in the setting of hindlimb ischemia (HLI) in *Adipoq* ^−/−^ mice [21] and further found that circulating adiponectin and CD34^+^ cell levels are associated in MI patients [9].

In the present study, we have determined the effects of adiponectin knockout on the mobilization of CACs in the setting of AMI. Secondly, we have examined the changes in expression of chemokines and adhesion molecules known to be implicated in ischemia-directed homing of CACs. In summary, we provide new evidence that adiponectin deficiency results in a diminished mobilization and homing capacity of CACs, leading to a reduction in neovascularization capacity. Clinically, these effects may translate into myocardial scar extension, impairment of myocardial function, and a higher degree of myocardial remodeling. 

## 2. Methods

### 2.1. Animals

National and European principles of laboratory animal care were followed. All animal experimental procedures were approved by the Animal Care and Use Committee of the University of Antwerp (Permit Number 2008-03). WT C57BL/6 and *Adipoq* ^−/−^ mice with deletion of exon 2 (translation initiation codon) of the adiponectin gene (strain name B6.129-*Adipoq* ^tm1Chan^/J) [22] were purchased from The Jackson Laboratory (Bar Harbor, Main, CA, USA). For all experiments, mice were housed in individually ventilated cages (IVC system) under pathogen-free conditions, in a normal day-night cycle (12/12) with free access to food and water. All mice used in the experiments were 4- to 5-month-old males.

### 2.2. Myocardial Infarction Mouse Model and Sample Collection

MI was induced by ligation of the left anterior descending (LAD) coronary artery in WT (*n* = 10) and *Adipoq* ^−/−^ (*n* = 7) mice. In brief, mice were anesthetized (Avertin 0.25 mg/g, intraperitoneally), intubated using a 22 G intravenous catheter, and mechanically ventilated with a small rodent ventilator (MiniVent type 845, Harvard Apparatus, ventilation at 10 *μ*L/g, 180 breaths/min, 2 cm H_2_O positive end-expiratory pressure). A left parasternal thoracotomy was performed transecting ribs 4 and 5. After adequate exposure of the heart, the pericardium was cleaved and the LAD was ligated approximately 2 mm below the left atrial appendage using 7-0 polypropylene sutures (Pronova BV-1, Ethicon, Johnson & Johnson). Successful LAD ligation was evidenced by white discoloration of the myocardium, elevation of the ST segment on electrocardiographic monitoring, and visual identification of the ligated artery in the infarction zone. Age-matched control mice were sham-operated (*n* = 8), that is, without tightening of the LAD ligature. Subsequently, the operation wound was closed in layers; mice were weaned from ventilation, extubated, and placed under a heat source until full recovery. Blood and tissue sampling were performed at 1 week after AMI, in accordance with the HLI experiments of Shibata et al. [21]. Mice were anesthetized and blood sampling was performed by direct intracavitary puncture in heparin precoated tubes. Specimens of the infarct border zone were harvested, flash-frozen in liquid nitrogen, and stored at −80°C for later use. Likewise, in the sham-operated mice, corresponding noninfarcted tissue specimens of the left ventricular free wall were collected and stored. A cross-section of the heart, taken 1 mm below the ligature, was fixed in paraformaldehyde (4%) and embedded in paraffin (PFPE) until histological analysis. BM was harvested by flushing both femurs with sterile phosphate buffered saline into heparin pre-coated tubes.

### 2.3. Echocardiography

Transthoracic echocardiography (AplioXV, 13 MHz linear probe, Toshiba) was performed on anesthetized mice just before the induction of AMI and at day 7 after AMI, respectively. Left ventricular end-systolic (LVESD) and end-diastolic (LVEDD) diameters and anterior and posterior wall thickness were measured at the midpapillary muscle level. Fractional shortening was calculated as ((LVEDD − LVESD)/LVEDD) × 100.

### 2.4. Tissue Homogenization, RNA Extraction, and Quality, cDNA Synthesis

Tissues were homogenized with an OmniTH tissue homogenizer (Mettler-Toledo). RNA was isolated using the RNeasy minifibrous tissue kit (Qiagen) following the manufacturer's instructions. On-column DNAse treatment (Qiagen) was used to remove contaminating DNA leftovers. RNA concentration and purity were analyzed using Nanodrop spectrophotometer (Nanodrop technologies) readings at 260 and 280 nm. Assessment of RNA integrity was done using Agilent 2100 Bioanalyzer (Agilent Technologies). Complementary DNA (cDNA) was synthesized by the transcriptor first-strand cDNA synthesis kit (Roche) according to the manufacturer's instructions and using a combination of random hexamer and oligo (dT) primers for reverse transcription. Reverse transcription was performed at 55°C for 30 minutes, followed by 5 minutes of incubation at 85°C to inactivate the reverse transcriptase enzyme. cDNA samples were placed on ice and stored at −20°C until further use. 

### 2.5. qPCR

Taqman gene expression assays (Applied Biosystems) were used for qPCR analysis on a LightCycler 480 instrument (Roche). All primers (see Table S1 in supplementary materials available online at http://dx.doi.org/10.1155/2013/260156) were designed to be intron spanning. qPCR was performed using the LightCycler Taqman Master Mix (Roche) in a final reaction volume of 20 *μ*L. We used the geNorm algorithm [23] to determine an optimal combination of reference genes for internal normalization (i.e., *Hprt*, *Tpt1*, and *Tbp*). Control of the KO status of *Adipoq* ^−/−^ mice was performed by qPCR using specific primers to detect the defective *Adipoq* gene (WT forward primer: 5′-TGGATGCTGCCATGTTCCCAT-3′; WT reverse primer: 5′-CTTGTGTCTGTGTCTAG GCCTT-3′; *Adipoq* ^−/−^ reverse primer: 5′-CTCCAGACTGCCTTGGGA-3′). All qPCR reactions were carried out as follows: after an initial denaturation-activation step at 95°C for 10 min, amplifications consisted of 45 cycles of denaturation at 95°C for 10 s, annealing at 60°C for 30 s and measurement of fluorescence at 72°C for 1 s. Cycle number (Cq) was measured using the baseline-independent second derivative maximum method. Normalized relative gene expression was determined by the *E* ^−ΔΔCq^ method. Assay efficiency (*E*) was measured by serial dilution of cDNA of pooled samples based on the slope of the standard dilution curve (*E* = 10^(−1/slope)^ − 1).

### 2.6. Histology and Immunohistochemical Analysis

PFPE tissues were used for morphometric analysis of infarct size (hematoxylin-eosin staining) and for analysis of capillary density. To this end, 5-*μ*m-thick sections were stained overnight with rabbit anti-mouse monoclonal anti-CD31 antibody (Abcam, ab56299) and subsequently conjugated with a secondary donkey anti-rabbit biotin-conjugated antibody (Jackson ImmunoResearch Laboratories) and a tertiary streptavidin HRP-conjugated antibody (Becton-Dickinson). Signals were revealed with the 3,3′-diaminobenzidine (DAB) tetrahydrochloride Chromogen System (DAKO). Images were captured with a Zeiss Axiophot microscope equipped with an Olympus DP70 camera and further processed in Adobe Photoshop to create composite images. Morphometric analysis measuring left ventricular and MI circumference at the midpapillary level was performed on composite microscopic images using the Cell^P^ image analysis software. 

### 2.7. Flow Cytometry

BM was suspended in PBS with heparin, filtered over a 40 *μ*m nylon mesh (Becton-Dickinson). White blood cell concentrations of the BM suspension and whole PB samples were determined using a hemocytometer (Micros 60, Horiba ABX). 10^6^ white blood cells were dispended in 1 mL wash solution (PBS supplemented with 0.5% BSA and 0.05% NaN_3_) and preincubated with rat anti-mouse FcR blocker (CD16/CD32) (BD Pharmingen) for 10 minutes. Hereafter, cells were further incubated in the dark for 30 minutes at room temperature using the following antibodies: APC-conjugated mouse lineage (lin) cocktail (BD Pharmingen) and PE-conjugated anti-mouse stem cell antigen-1 (Sca-1) (BD Pharmingen). Red blood cells were lysed in ammoniumchloride buffer (StemCell Technologies) for 10 minutes, followed by two 5 min wash steps. 5 × 10^5^ events were counted with a FACSCantoII flow cytometer system (Becton-Dickinson). Unstained and fluorescence-minus-one (FMO) controls were used to determine autofluorescence background signals and allowed for the appropriate gating of positive events. The forward scatter/side scatter (FSC/SSC) plot was used to gate out the mononuclear cell population. Cell aggregates and cell debris were banned on the FSC-height/FSC-area scatter plot.

### 2.8. Statistical Analysis

Statistical analysis was performed in PASW statistics 18 (IBM Corp.). Graphs were created in GraphPad Prizm. Tabular data are expressed as mean ± SEM. Unless otherwise specified, nonparametric tests (Mann-Whitney *U*-test and Kruskal-Wallis test for comparison of two or more than two groups and Spearman's rank correlation coefficient for correlation analysis, resp.) were used because of nonnormality of data subsets. A two-sided *P* value of <0.05 indicated statistical significance.

## 3. Results

### 3.1. Baseline Characteristics

Overall, we observed larger infarctions one week after AMI in *Adipoq* ^−/−^ compared to WT mice (61.5% versus 49.4%, resp.) (Supplementary Figure S1) and more signs of heart failure (HW/TL 9.5 versus 8.6, resp.) and reduced LV function on echocardiography (fractional shortening 28.2% versus 35.0%, resp.) in *Adipoq* ^−/−^ compared to WT mice. Unfortunately, because of the small sample size and high variance, these differences did not reach statistical significance (Table 1). Baseline morphometric and echocardiographic characteristics of WT sham and *Adipoq* ^−/−^ sham mice were comparable (data not shown).

### 3.2. Effects of Adiponectin Knockout on PC Numbers

**Figure 1:**
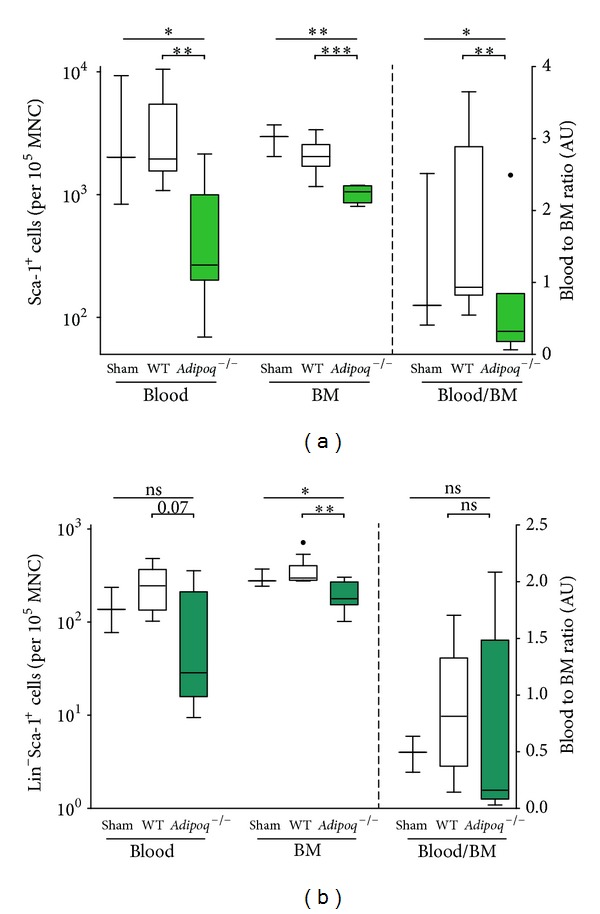
PC number in blood and BM and influence of *Adipoq* ^−/−^ Different progenitor populations were investigated by flow cytometry. Compared to WT animals, the numbers of bone marrow-residing Sca-1^+^ and Lin^−^Sca-1^+^ subpopulations (expressed per 10^5^ cells, depicted on the left axis) were markedly diminished in *Adipoq* ^−/−^ conditions. Sca-1^+^ and Lin^−^Sca-1^+^ progenitors in the blood showed a similar trend. Additionally, the B/BM ratio (%, depicted on the right axis) was reduced for Sca-1^+^, which could indicate an additional mobilization deficit for Sca-1^+^ cells under *Adipoq* ^−/−^ conditions. *P < 0.05; **P < 0.01; ***P < 0.001; ns: not statistically significant; dots represent outliers.

We used flowcytometry to determine the number of Sca-1^+^ and Lin^−^Sca-1^+^ PCs in BM and PB one week after AMI. In *Adipoq* ^−/−^ mice, the BM-residing Sca-1^+^ and Lin^−^Sca-1^+^ PC populations were markedly reduced compared to those in WT mice (*P* < 0.001; *P* = 0.003, resp.). PB-mobilized circulating Sca-1^+^ PCs were diminished in *Adipoq* ^−/−^ mice (*P* = 0.003) and a similar trend was visible for the Lin^−^Sca-1^+^ fraction (*P* = 0.07). Moreover, reduction in the proportion of Sca-1^+^ PCs in PB compared to BM in *Adipoq* ^−/−^ mice (*P* = 0.01), suggested a mobilization defect under adiponectin-deficient conditions, in addition to the absolute reduction of the available BM PC pool (Figure 1). A similar reduction in PC number in PB and BM as evidenced for *Adipoq* ^−/−^ after AMI was found in *Adipoq* ^−/−^ sham mice (data not shown).

### 3.3. Effects of Adiponectin Knockout on CAC Homing

**Figure 2:**
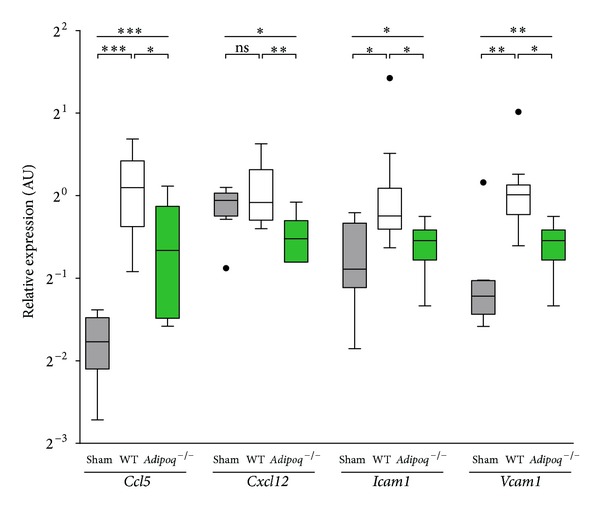
Gene expression of homing factors. Relative expression levels of chemokines and adhesion molecules were determined in sham (grey), WT (white), and *Adipoq* ^−/−^ (green) animals 1 w after AMI. qPCR experiments revealed a markedly blunted upregulation of several chemokines and both *Icam1* and *Vcam1* in *Adipoq* ^−/−^ conditions. *P < 0.05; **P < 0.01; ***P < 0.001; ns: not statistically significant; dots represent outliers.

To examine the effects of adiponectin deficiency on peripheral CAC homing in the setting of MI, we determined the genetic expression of several hypoxia-induced chemokines and adhesion molecules known to be implicated in CAC trafficking. mRNA levels of *Ccl5* and *Cxcl12* were found to be significantly downregulated in the MI border zone of *Adipoq* ^−/−^ mice compared to WT (*P* = 0.025; *P* = 0.005, resp.). Additionally, the mRNA expression of two integrin ligands implicated in ischemia-directed progenitor cell homing, *Icam1* (*P* = 0.010) and *Vcam1* (*P* = 0.014), was attenuated in the MI border zone of *Adipoq* ^−/−^ mice (Figure 2). This reduction in the expression of chemokines and vascular adhesion molecules in the MI border zone in *Adipoq* ^−/−^ mice could indicate a mobilization and homing deficit for BM-residing PCs or CACs, respectively. mRNA levels of WT sham and *Adipoq* ^−/−^ sham mice were comparable, except for hif1a, which was higher in the *Adipoq* ^−/−^ sham group (data not shown). 

**Figure 3:**
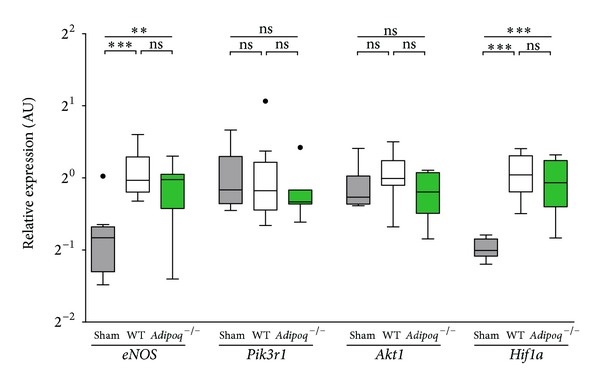
Gene expressions of the PI3 K/AKT/eNOS and HIF-1 pathways. Relative gene expression levels of *eNOS*, *Pik3r1*, *Akt1*, and *Hif1a* were determined in sham (grey), WT (white), and *Adipoq* ^−/−^ (green) animals 1 w post-AMI. *P < 0.05; **P < 0.01; ***P < 0.001; ns: not statistically significant; dots represent outliers.

Mechanistically, we observed several strong positive correlations in the expression pattern of homing factors, *eNOS* and *Hif1a*, in the MI border zone. Interestingly, these associations were only found in WT mice, in which a positive correlation was observed between *eNOS* and the expression of the regulatory subunit of PI3 K (*Pik3r1*) (*P* < 0.001), *eNOS*, and *Hif1a* (*P* = 0.048) and *eNOS* and homing factors situated downstream of HIF-1, such as *Cxcl12* (*P* = 0.022) and *Icam1* (*P* = 0.025). Moreover, in WT mice, *Cxcl12* induction was associated with the upregulation of *Hif1a* (*P* = 0.033), *Icam1* (*P* < 0.001), and *Vcam1* (*P* < 0.001) and with *Pik3r1* (*P* = 0.029). Conversely, no significant correlations were found in *Adipoq* ^−/−^ mice. Although our data did not indicate a significant reduction in the average *eNOS* or *Hif1a* expression between WT and *Adipoq* ^−/−^ mice (Figure 3), the previously-mentioned associations seem to indicate that the PI3 K/AKT/eNOS pathway and the HIF-1 hypoxia-signaling pathway are coregulated in WT but not in *Adipoq* ^−/−^ mice after AMI.

### 3.4. Effects of Adiponectin Knockout on Neovascularization after AMI

**Figure 4:**
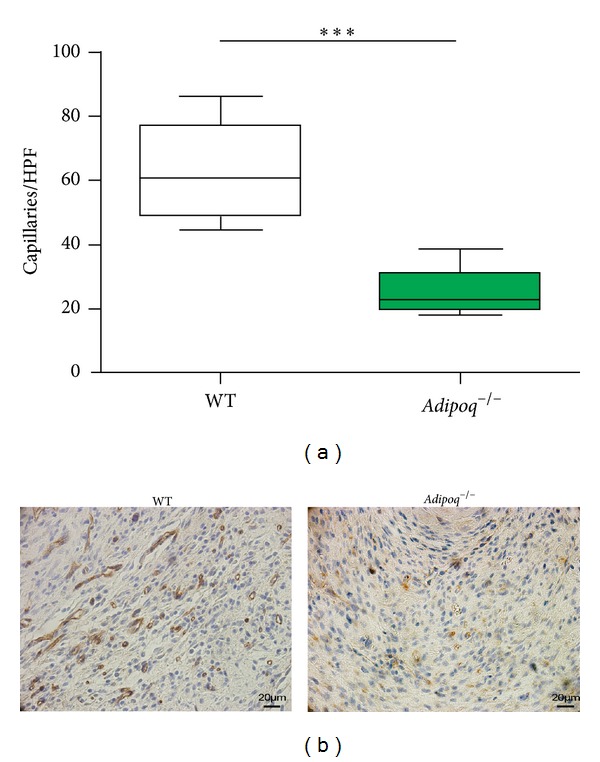
Capillary density and the influence of adiponectin deficiency. The number of capillary structures in the infarction border zone was lower in *Adipoq* ^−/−^ than in WT mice 1 w post-AMI. HPF: high-power field (400x); ***P < 0.001.

The capillary density in the MI border zone was increased over twofold in WT mice compared to *Adipoq* ^−/−^ mice (*P* < 0.001), pointing to a neovascularization deficit in adiponectin-deficient conditions (Figure 4). Overall, the increase in capillary density was positively correlated with Sca-1^+^ PC numbers in BM (*P* = 0.01) and PB (*P* = 0.005), and with mRNA levels of homing factors *Cxcl12* (*P* = 0.013), *Icam1* (*P* = 0.034), and *Vcam1* (*P* = 0.014), which could be indicative of a close interrelationship between CAC mobilization, homing, and neovascularization after AMI. Conversely, capillary density was negatively correlated with parameters of infarct remodeling, such as tibia length-corrected heart weight (*P* = 0.041).

## 4. Discussion

In the present study, we investigated the effects of adiponectin on CAC mobilization, peripheral homing signals, and neovascularization in the setting of AMI. One week after AMI, *Adipoq* ^−/−^ mice displayed a reduction in the number of BM-residing and circulating angiogenic cells, coinciding with a downregulated gene expression of the chemotactic factors *Cxcl12* and *Ccl5*, and the vascular adhesion molecules *Icam1* and *Vcam1*. The decreased BM reserve, mobilization and homing capacity of CACs could be important factors contributing to the decreased neovascularization capacity that was observed under adiponectin deficient conditions.

The mobilization of BM-derived CACs into the circulation is primarily driven by hypoxia-induced growth factors and cytokines [24]. In conditions associated with decreased oxygen tension, the *α* subunit of hypoxia-inducible factor 1, HIF-1*α*, is upregulated at both the mRNA and the protein level [25] and translocates to the nucleus, where it promotes angiogenesis and vascular remodeling by enhancing the expression of multiple proangiogenic factors, such as CXCL12 [26], placental growth factor [27], and VEGF [28]. CXCL12 is considered to be the main chemokine involved in ischemia-directed CAC mobilization and homing, occurring along a (reversed) BM-to-PB CXCL12 gradient [29]. In microvascular endothelium, CXCL12 activates the PI3 K/AKT/eNOS signaling pathway, which results in phosphorylation of eNOS and a concomitant increase in NO production [30]. eNOS has been reported to be essential for CAC mobilization from the BM [31, 32] by upregulation of matrix metalloproteinase 9 (MMP9) [33]. 

Our findings confirm both the lower pool of available CACs in the BM and the decreased mobilization of CACs under *Adipoq* ^−/−^ conditions. Similar results on CAC BM reserve and mobilization were reported by Shibata et al. [21] in the setting of HLI. We suggest that these effects are due to a defective activation of eNOS, thereby attenuating CAC biology. Chen et al. [19] already demonstrated that adiponectin is able to stimulate the production of NO in *in vitro* cultures of endothelial cells, a process that proved to be dependent on AMPK. In human umbilical vein endothelial cells, adiponectin has further been reported to induce AMPK phosphorylation, which stimulated phosphorylation of AKT in a PI3 K-dependent manner. In turn, phosphorylated AKT induced phosphorylation of eNOS, converting eNOS into its active form [15]. These studies suggest the existence of an adiponectin-induced AMPK/PI3 K/AKT/eNOS signaling pathway. In this regard, Tao et al. [14] confirmed the reduced presence of phosphorylated eNOS in the area at risk of IR injured cardiac tissues obtained from *Adipoq* ^−/−^ mice.

NO has been proven to stimulate HIF-1 signaling in a number of ways. First, inhibition of cyclic guanosine monophosphate (cGMP) degradation by the phosphodiesterase type 5 (PDE5) inhibitors sildenafil and vardenafil led to increased HIF-1*α* expression and enhanced blood flow recovery, capillary formation, and progenitor mobilization in an HLI mouse model. Because NO activates guanylate cyclases, which are the enzymes responsible for cGMP production, NO levels could have direct effects on HIF-1*α*. This hypothesis was confirmed in an HLI model in *eNOS* ^−/−^ mice, where eNOS deficiency resulted in the abrogation of the previously-mentioned effects of PDE5 inhibitors on capillary formation and HIF-1*α* protein expression [34]. These authors suggested that eNOS/NO were essential for cGMP-induced angiogenesis and upregulation of hypoxic signaling in ischemic tissues. A second mechanism by which eNOS and NO enhance HIF-1 activity consists in preventing the degradation of the HIF-1*α* subunit through inhibition of prolyl hydroxylases (PHDs), which tag HIF-1*α* for polyubiquitination and subsequent proteasomal degradation, a process which occurs rapidly under normoxic conditions [35]. Thirdly, both PHD inhibition [36] and the activation of the PI3 K/AKT signaling pathway [37] have been reported to stimulate the activity of NF*κ*B signaling. NF*κ*B has been proved to be a critical transcriptional activator of HIF-1*α* [38], indicating a high degree of crosstalk and overlap between inflammation and hypoxia-induced gene expression programs. 

Our study revealed several positive correlations between *eNOS*, *Hif1a*, and the expression of downstream homing factors in WT conditions. Interestingly, our data did not evidence these correlations in *Adipoq* ^−/−^ mice. Moreover, the gene expression of several homing factors and vascular adhesion molecules was downregulated in *Adipoq* ^−/−^ mice. This finding is interesting since adiponectin has earlier also been reported to suppress ICAM1 and VCAM1 expression *in vitro* [39] through inhibition of TNF*α*-induced NF*κ*B signaling [17]. These studies, however, did not investigate the specific situation of tissue ischemia but only reported the effects of adiponectin on TNF*α*-induced inflammation in endothelial cell cultures. The definite effect of adiponectin on endothelium under hypoxic conditions has yet to be determined in further studies. 

In conclusion, we report a decrease in capillary density in *Adipoq* ^−/−^ mice compared to WT mice, which coincided with a reduction in the number of BM-residing and circulating angiogenic cells and a downregulation in the expression of homing factors. Targeting adiponectin metabolism could be clinically relevant to enhancing hypoxia signaling and CAC homing in ischemic conditions.

## Supplementary Material

Taqman gene expression assay specifications are provided as an online data supplement.
Figure S1. Heart cross sections of WT vs. Adipoq−/− mice Upper row: WT (n=3). Lower row: *Adipoq* ^−/−^ (n = 3).

## Figures and Tables

**Table 1:** Clinical characteristics (mean ± SEM) at 1 w after AMI.

	Sham (*n* = 8)	WT (*n* = 10)	*Adipoq* ^−/−^ (*n* = 7)	*P* (WT versus *Adipoq* ^−/−^)
Age (weeks)	17.9 ± 1.2	21.0 ± 0.8	19.9 ± 0.1	0.475
HW (mg)	122.3 ± 3.8	153.7 ± 8.0	170.7 ± 10.0	0.133
HW/TL (g/m)	6.81 ± 0.20	8.64 ± 0.44	9.49 ± 0.55	0.133
LVEDD (mm)	3.25 ± 0.23	4.40 ± 0.16	4.30 ± 0.30	0.713
FS (%)	NA	35.0 ± 3.9	28.2 ± 4.7	0.315
Infarct size (%)	NA	49.4 ± 5.4	61.5 ± 6.4	0.193

Morphometric and echocardiographic parameters were not significantly different between WT and *Adipoq* ^−/−^ animals, although absolute values tend to show an increase in infarction size and post-AMI remodeling parameters in *Adipoq* ^−/−^ mice. HW: heart weight; TL: tibia length; LVEDD: left ventricular end-diastolic diameter; FS: fractional shortening; *Nppb*: natriuretic peptide B; AU: arbitrary units; NA: not available.
