# Supplementary material for: Adiponectin Deficiency Blunts Hypoxia-Induced Mobilization and Homing of Circulating Angiogenic Cells
Source: Stem Cells Int. 2013 Oct 29;2013:260156. doi: 10.1155/2013/260156 (PMC3830768; doi:10.1155/2013/260156)
Supplement: Supplementary file 1 — Taqman gene expression assay specifications are provided as an online data supplement. Figure S1. Heart cross sections of WT vs. Adipoq−/− mice Upper row: WT (n=3). Lower row: Adipoq −/− (n = 3). [file 260156.f1.docx]

SUPPLEMENTARY DATA

**Table S1. Gene list and qPCR assay information**

| **Gene** | **Full name/synonyms** | **Function** | **AB ID** |
| --- | --- | --- | --- |
| ***Akt1*** | v-Akt murine thymoma viral oncogene homolog 1 | Plays a key role in cell survival, insulin signaling and angiogenesis | Mm01331624_m1 |
| ***Ccl2*** | Chemokine (C-C motif) ligand 2  Syn: monocyte chemotactic protein-1 (MCP-1) | Chemotactic activity on cells of the monocytic lineage | Mm00441242_m1 |
| ***Ccl5*** | Chemokine (C-C motif) ligand 5  Syn: RANTES (Regulated upon Activation, Normal T-cell Expressed, and Secreted) | Recruits circulating monocytes and has anti-apoptotic effects on tissue macrophages | Mm01302428_m1 |
| ***Ccl7*** | Chemokine (C-C motif) ligand 7  Syn: monocyte-specific chemokine 3 (MCP-3) | Chemotactic activity on cells of the monocytic lineage | Mm00443113_m1 |
| ***Cxcl12*** | Chemokine (C-X-C motif) ligand 12  Syn: stromal cell-derived factor-1 (SDF-1) | Strongly chemotactic for lymphocytes and macrophages and important in hematopoietic stem cell homing | Mm00445552_m1 |
| ***Cxcr4*** | C-X-C chemokine receptor type 4 | Interacts with CXCL12 | Mm01292123_m1 |
| ***Hif1a*** | Hypoxia inducible factor 1, alpha subunit | Transcription factor regulating homeostatic responses to hypoxia, role in (tumor) angiogenesis and ischemic disease pathophysiology | Mm00468875_m1 |
| ***Icam1*** | Intercellular adhesion molecule 1 | Expressed on endothelial cells and immune cells. Binds to LFA-1 integrins | Mm00516023_m1 |
| ***Nos3*** | Nitric oxide synthase 3 (endothelial cell)  Syn: eNOS | Catalyzes the generation of nitric oxide, mediator of cardiovascular homeostasis. Vasodilative, anti-atherosclerotic and anti-apoptotic properties | Mm00435204_m1 |
| ***Nppb*** | Natriuretic peptide B | Upregulated in heart failure conditions. Key role in natriuresis and vasorelaxation | Mm01255770_g1 |
| ***Pik3r1*** | Phosphatidylinositol 3-kinase regulatory subunit alpha | Encodes the 85 kD regulatory subunit of PI3K | Mm01282781_m1 |
| ***Vcam1*** | Vascular cell adhesion molecule 1 | Expressed on stimulated endothelial cells. Interacts with VLA-4 | Mm00449197_m1 |

AB ID: Applied biosystems Taqman® gene expression assay ID.


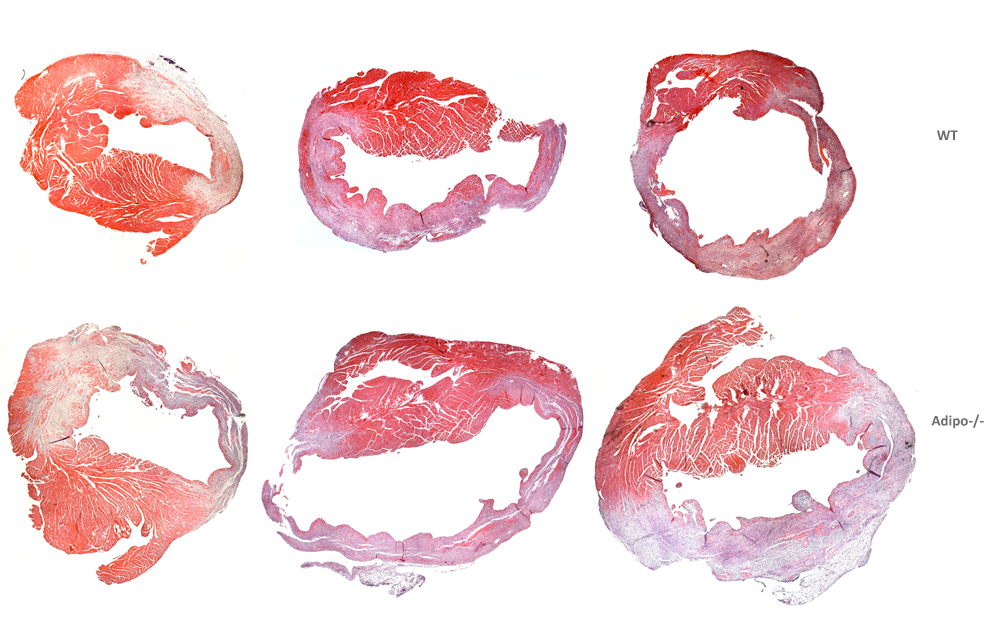


**Figure S1. Heart cross sections of WT vs. *Adipoq^-/-^* mice**

Upper row: WT (n=3). Lower row: *Adipoq^-/-^* (n=3)
